# Bound states in the continuum and long-lived electronic resonances in two-tangent loops cavity

**DOI:** 10.1038/s41598-025-26444-9

**Published:** 2025-11-23

**Authors:** Eman A. Abdel-Ghaffar, Leonard Dobrzyński, Housni Al-Wahsh, Abdellatif Akjouj

**Affiliations:** 1 Electrical Engineering Department, Faculty of Engineering, Benha University, Shoubra, Cairo 11629 Egypt; 2 Département de Physique, Institut d’Electronique, de Microélectronique et de Nanotechnologie (IEMN), UMR CNRS 8520, Université de Lille, 59655 Villeneuve d’Ascq Cédex, France; 3 Engineering Mathematics and Physics Department, Faculty of Engineering, Benha University, Shoubra, Cairo 11629 Egypt

**Keywords:** Bound states in the continuum, Fano resonances, Transmittance, Near-perfect absorption, State phase, Filtering, Nanoscience and technology, Optics and photonics, Physics

## Abstract

Bound states in the continuum (BICs) has emerged as a significant research focus in electronics due to its exceptionally high quality factor (Q-factor). BICs (known also as trapped modes) are not observable from the spectrum due to their non-radiative property. However, they can exist only under a specific choice of the materials or geometrical parameters of the structure. In this paper a BIC eigenfunction is defined to be strictly localized within a subspace of the cavity structure under study and has no leakage behaviour. Its eigen wavelength can be within state continua. BICs and long-lived resonances (LLR) have become a unique way to produce the extreme confinement of electronic waves. We present a theoretical and numerical demonstration of semi-infinite bound states in the continuum (SIBICs) and LLR in a two ring-like electronic micro-cavity coupled to two electronic rib/ridge wave-guides, together with their existence conditions. This structure is composed of two tangent closed loops of lengths $L_1$ and $L_2$, and two semi-infinite leads. SIBICs are localized in a semi-infinite subspace domain induced transmission zeros. Other induce transmission ones in the middle of long-lived resonances. The BICs correspond to localized resonances of infinite lifetime inside the cavity, without any leakage into the surrounding leads. When BICs exist within state continua, they induce Fano resonances exhibiting sharp peaks in the transmittance spectra and in the variation of the density of states (VADOS) for specific values of the geometrical parameters $L_1$ and $L_2$. We demonstrate that the condition for the existence of the BICs is to make the lengths $L_1$ and $L_2$ commensurate with each other. This enables to control the resonances by engineering these lengths. Finally, such a two-tangent loops cavity can be designed to realize near-perfect absorption for some frequencies. The results obtained take due account of the state number conservation between the final system and the reference one. This conservation rule enables to find all the states of the final system and among them the BIC ones. The analytical results are obtained by means of the Green’s function technique. The cavity structure and the LLR presented in this work may have potential applications due to their high sensitivities to weak perturbations, in particular in sensing and wave filtering.

## Introduction

The interaction of electronic waves with scatterers in a composite material generates resonances and anti-resonances in the transmittance spectra^1–5^. These important phenomena, including Fano resonances, have been demonstrated in electronic waveguides^6,7^. In Fano resonance, the peak is followed by anti-resonance in a narrow frequency range, giving rise to an asymmetrical line profile shape. When the resonance falls between two anti-resonances, Fano resonance acts like a LLR^8–11^. These resonances are the result of a discrete localized mode interacting with a continuum of propagating modes, which can transform a transparent system into an opaque one in a narrow frequency range^12^ (for a complete study of resonant modes see e.g.^13^).

In recent years BICs have brought significant attention due to their important design principle to create systems that can enhance light-matter interaction^14^. These non-radiating modes are localized within a continuum of extended modes, yet they remain highly confined with an infinite lifetime and quality factor (Q factor) in lossless systems. Hence, BICs remain well confined in some finite parts of the system (subsystem), even though they coexist with a continuous spectrum of outgoing waves that can transport energy away. BIC and Fano resonance, or LLR, i.e. transmittance one (reflectance zero) squeezed between two transmittance zeros (two reflectance ones) were observed in quantum systems^8,15,16^; however, it was shown that these resonances can be extended to other systems such as photonics^17–22^, acoustics^23,24^, magnonics^25^, electronics^26,27^, plasmonic nanostructures^28–30^, metasurfaces^31,32^, and fiber Bragg gratings^33^. Increasing interest in BICs results from their potential use in several applications such as filters^34,35^, sensors^36,37^ and lasers^38^. BICs are categorized into several mechanisms according to their discovery origin^14^, including; Fabry-Perot (FP) BICs^12,39–41^, Freidrich-Wintgen BICs^12,42–45^, symmetry-protected BICs^31,46–48^ and accidental BICs^49–51^. These BICs have been investigated both theoretically and experimentally in different physical systems^52–56^. BICs are non-radiative modes with vanishing spectral linewidth, this property makes them non-observable in the spectrum, they can only exist under specific choice of the geometrical parameters (or the material) of the structure under study. By slightly detuning these parameters from the BIC conditions, one can have a LLR with a finite width. BICs has also introduced (and become a perfect scar) in the theory of quantum graphs. The theory provides a powerful framework to understand BICs. The topological and geometrical characteristics of the theory (specially the boundary conditions at the vertices) allow for the emergence of BICs, see e.g.^57^.

A confined state is strictly localized within a real space domain (e.g. real linear path). Its strict localization is due to its eigen function zeros, called robust zeros and its connection with its outside space only through these robust zeros. When this path has a finite length and its wavelength falls within an eigenstate bulk band, the confined path state is a bound in continuum (BIC) state^16^. When this path has a semi-infinite length, the confined path state is a SIBIC state^58^.

Any state with eigenfunction zero at the connection point can not interact with another one^59–61^. When the leads are attached to the cavity structure, some final states may be BICs, or SIBICs. This work presents BICs and SIBICs inducing transmission zeros (ones) and LLR. The results are obtained utilizing the interface response theory, used to solve the problem of the propagation of electronic waves in a structure with different connection points^62^. This method enables also to deduce the transmittance, the transmittance phase, the phase time, the state phase shifts and the variations of the density of states. The results in this work, takes due consideration of the state number conservation rule between the final system and the reference one constituted by the independent closed loops (of lengths $L_1$ and $L_2$) and the semi-infinite leads. This rule enables us to find all the states of the final system and in particular the BICs ones^60^.

## Inverse surface Green’s functions of the constituents

We report here the expression of the Green’s function of a homogeneous isotropic infinite medium. For the sake of simplicity, we restrict ourselves to homogeneous guides. We give also the inverse of the surface Green’s function for the semi-infinite guide with a free surface and for the finite guide of length $L_1$ (or $L_2$).

### Infinite guide

**Fig. 1:**
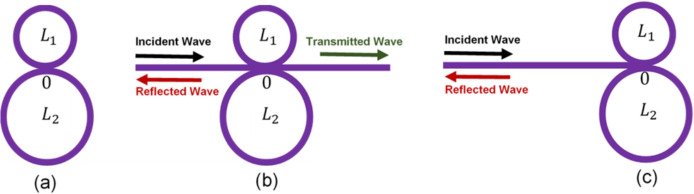
(**a**) The two tangent loops cavity studied in this work. The cavity made out of two tangent loops of lengths $L_1$ and $L_2$. When closing the guide $L_1$ at space point 0, one connects to it the 0 end of the guide of length $L_2$ (figure a). Finally two semi-infinite leads are connected to the space point 0 (figure **b**). The space point 0 is the point of connection of the two semi-infinite leads with the two loops, $L_1$ and $L_2$, constituting the cavity. This is why we call it *interface point*. The *interface space* for this cavity structure is the point 0. This interface space is written shortly as: $M=\left\{ 0\right\}$. Let us also mention that the Kirchoff-Neumann boundary condition is used at the interface point 0. (**c**) The two loops cavity structure is attached only to one port from one side.

We describe the electronic wave propagation in the frame of free particle model in which $E=(\hbar ^2 k^2/2m)+V$, where *m*, *V*,  and *k* refer respectively to the effective mass, a constant potential and a wave vector. In this paper, we focus on homogeneous structures where all media (the two loops and the semi-infinite leads, see Fig. 1) are made of the same material, namely, GaAs. The material parameters are then $V=0.0$ meV and $m=0.067~m_0$, where $m_0$ is the free electron mass. The time independent Schrödinger equation for standing electronic waves is^63^

1 $$\begin{aligned} \left( \frac{d^2}{d x^2}-k^2\right) \psi (x)=0, \end{aligned}$$

where *x* is the space position along the structure and $k=\frac{1}{\hbar }\sqrt{2mE}$. The response function $G(x, x' )$ of this infinite guide is defined by

2 $$\begin{aligned} \left( \frac{d^2}{d x^2}-k^2\right) G(x, x' )=\delta (x-x'), \end{aligned}$$

the corresponding response function is

3 $$\begin{aligned} G(x, x' )=\frac{e^{ik\mid x-x'\mid }}{2ik}. \end{aligned}$$

where $\delta$ stands for the Dirac delta distribution, also known as the unit impulse and $i=\sqrt{-1}$.

### Semi-infinite guide

In our previous work^2^ we discussed in details Green’s function for semi-infinite guide. We demonstrated that, for a semi-infinite guide with a “free surface” located at the position $x = 0$ in the direction *Ox* of the Cartesian coordinates, the inverse surface Green’s function is given by^2,64^

4 $$\begin{aligned} g^{-1}_s(M,M)=g^{-1}_s(0,0)=ik, \end{aligned}$$

### Finite guide

In our previous work^2,64^ we illustrated that, for a finite guide of length $L_j (j=1,2)$ bounded by two free surfaces located on $x = 0$ and $x = L_j$ in the direction *Ox* of the Cartesian coordinates system, the surface Green’s function is given by;

5 $$\begin{aligned} g_{L_j}(M,M)= \frac{1}{k S(L_j)} \left( \begin{array}{cc} C(L_j)& 1 \\ 1& C(L_j) \\ \end{array} \right) , \end{aligned}$$

where $C(L_j) = \cos (kL_j)$ and $S(L_j) = \sin (kL_j), j=1,2$. The elements of this matrix gives the interface response functions $g(0,0)=g(L_j, L_j)=C(L_j)/(k S(L_j)), g(0,L_j)= g(L_j, 0)=1/(k S(L_j)), j=1,2$.

#### One loop

A mono-mode guide of length $L_j (j=1,2)$ is a basic building block of any linear material, device and system. When closing the guide by superimposing the ends 0 and $L_j$, the inverse of the interface response function at the interface point 0 (using Eq. 5) is given by

6 $$\begin{aligned} g^{-1}(0,0)=-2k\dfrac{C(L_j)}{S(L_j)}+2k\dfrac{1}{S(L_j)}=2k\dfrac{S(L_j/2)}{C(L_j/2)}. \end{aligned}$$

### Two loop states

Consider now a reference system made out of two guides of lengths $L_1$ and $L_2$. When the four ends of these two guides are superposed on the same space site point 0, we obtain the two tangent closed loops presented in Fig. 1a. The inverse of the response function element g(0, 0) of these two tangent loops without the the semi-infinite leads is

7 $$\begin{aligned} [g(0,0)]^{-1} =-2 k\left( \frac{C(L_1)}{S(L_1)} +\frac{C(L_2)}{S(L_2)}- \frac{1}{S(L_1)}-\frac{1}{S(L_2)}\right) . \end{aligned}$$

A simple algebra leads to:

8 $$\begin{aligned} \begin{aligned} [g(0,0)]^{-1} =2 k \left( \frac{ S(L_1/2)C(L_2/2)+S(L_2/2)C(L_1/2)}{C(L_1/2)C(L_2/2)}\right) =\frac{ 2 k S(L)}{C(L_1/2)C(L_2/2)},~~~~~~~~~~~~~~~~~~~~~~~~~~~~~~~~ \end{aligned} \end{aligned}$$

where $$L=(L_1+L_2)/2$$.

For each finite guide of length $L_j (j=1,2)$, the discrete states are given by the poles of the Green’s function, namely $kS(L_j) = 0$ (see Eq. 5), therefore the initial states of the system composed of the two independent guides $L_1$ and $L_2$ are given by $(k S(L_1))(k S(L_2)) = 0$. We have also for each finite guide (using Eq. 5), the determinant of the surface Green’s function is given by $\mid g^{-1}_{L_j} (M,M)\mid =-k^2$. So the final states of the structure presented in Fig. 1a are given by the state number conservation and the state phase shift^60^ to be:

9 $$\begin{aligned} \left[ k S(L_1) \right] \left[ k S(L_2)\right] \mid [g(0,0)]^{-1} \mid \left[ \dfrac{1}{(-k^2)^2}\right] =0. \end{aligned}$$

that is,

10 $$\begin{aligned} \frac{1}{k} S(L_1/2)S(L_2/2)S(L) = 0. \end{aligned}$$

Equation (10) provides all the states of the finite two loop system, given by Fig. 1a. Once one adds the two semi-infinite leads (Fig. 1b), an infinite continuum of other states are added, represented by the 2*ik* of Eq. (4). Most of these states contribute to build the wave packets forming the long lived resonances close to the BIC states. With the help of Eqs. (8) and (10) one can find the eigenvector values *u* for particular states of the final system from

11 $$\begin{aligned} [g(0,0)]^{-1}u= 0. \end{aligned}$$

For the states defined by $S(L) = 0$, i.e., $kL/2\pi = n/2$, where $n = 0, 1, 2,...$ we get $u = 1$. For the states defined by $C(L_1/2) = 0$ (or $C(L_2/2) = 0$), i.e., $kL/2\pi = (n+0.5) L/L_1$ (or $kL/2\pi = (n+0.5) L/L_2$), where $n = 0, 1, 2,...$ we get $u = 0$. For the states defined by $S(L_1/2) = 0$ and $S(L_2/2) \ne 0$ (or $S(L_2/2) = 0$ and $S(L_1/2) \ne 0$) i.e., $kL/2\pi = n L/L_1$ (or $kL/2\pi = n L/L_2$), where $n = 0, 1, 2,...$ we get $u = 0$.

According to the BIC state theorems given in our previous work^58^, once the two leads are connected (Fig. 1b), the above eigenvector zeros are the signatures of BIC (or SIBIC) states and transmission zeros. Also the eigenvector ones are the signatures of transmission ones, falling in between the transmissions zeros and being then the tops of LLR.

## Transmission and reflection functions

The structure presented in Fig. 1b is composed of, two tangent loops of lengths $L_1$ and $L_2$, inserted between two semi-infinite leads. The inverse of Green’s function of the whole system is given by a linear superposition of the Green’s functions of its constituents given above (Eqs. 4 and 8) in the interface space $M = \left\{ 0 \right\}$, namely^65^:

12 $$\begin{aligned} [g(0,0)]^{-1} = 2k\left( \frac{ S(L)}{C(L_1/2)C(L_2/2)}+i\right) . \end{aligned}$$

Let us consider an incident wave $U(x) = e^{-ikx}$ launched in the left semi-infinite lead (Fig. 1b). From Eq. (12), one can obtain the transmission function in the right semi-infinite lead, namely, $t = -2ik g(0,0)$, or equivalently:

13 $$\begin{aligned} t = \frac{-iC(L_1/2)C(L_2/2)}{S(L) +i C(L_1/2)C(L_2/2)}. \end{aligned}$$

The transmittance *T*($=\mid t \mid ^2$) is

14 $$\begin{aligned} T = \frac{C^2(L_1/2)C^2(L_2/2)}{S^2(L) + C^2(L_1/2)C^2(L_2/2)}. \end{aligned}$$

In the same way, the reflection function in the left semi-infinite lead is given by $r=-1+2ik g(0,0)$ and the reflectance *R*($=\mid r \mid ^2$) is

15 $$\begin{aligned} R = \frac{S^2(L)}{S^2(L) + C^2(L_1/2)C^2(L_2/2)}. \end{aligned}$$

From Eqs. (14) and (15), one can easily check (in the absence of loss) the conservation law $R + T = 1$.

### BIC states

The two tangent loops structure (Fig. 1) can exhibit BICs and Fano resonances. BICs are described as resonances with zero widths in the transmittance and density of states spectra. These states can occur only under specific geometrical lengths $L_1$ and $L_2$. When departing slightly from the BIC conditions, they transform to specific Fano resonances that are characterized by a zero Fano parameter. It is well known that the eigenmodes of the structure presented in Fig. 1b are given by the poles of the transmission function *t* (Eq. 13) or equivalently by the poles of the Green’s function (Eq. 12), namely

16 $$\begin{aligned} S(L) +i C(L_1/2)C(L_2/2)=0. \end{aligned}$$

The above equation is a complex quantity. Its real part gives the position of the resonances in transmission and density of states, whereas its imaginary part is related to the width of the resonance and also here to the transmission active states. In general, it is not easy to simultaneously cancel the real and imaginary parts of this equation at the same frequency. This will correspond to a bound state falling in the continuum of states. In order to avoid the divergence of *t*, then its numerator should also vanish in such a way that *t* becomes finite. These two conditions can be fulfilled only if $C(L_1/2)=0$ and $C(L_2/2)=0$. A simple algebra leads to the following condition

17 $$\begin{aligned} \dfrac{L_1}{L_2}=\dfrac{p}{q}, \end{aligned}$$

where *p* and *q* are odd integers. This leads to the conclusion that $L_1$ and $L_2$ should be commensurate with each other. Let us define a unit length $L_0$ such that the two loops lengths $L_1$ and $L_2$ are multiple of $L_0$ (i.e. $L_1=p L_0$ and $L_2=q L_0$). Therefore Eq. (16) can be written as

18 $$\begin{aligned} \begin{aligned} S(p L_0/2) C(q L_0/2) +S(q L_0/2)C(p L_0/2) + i C(p L_0/2) C(q L_0/2)=0. \end{aligned} \end{aligned}$$

or equivalently

19 $$\begin{aligned} \begin{aligned} S(p L') T_q [C(L')] +S(q L') T_p [C(L')] + i T_p [C(L')] T_q [C(L')]=0. \end{aligned} \end{aligned}$$

**Fig. 2:**
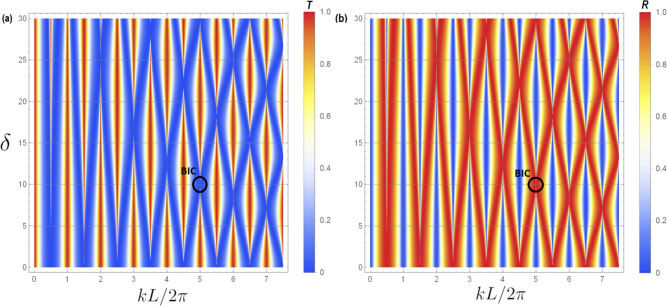
For the cavity structure presented in Fig. 1b the above plots shows the variation of transmittance (**a**) and reflectance (**b**) (with color scale) versus the reduced wave vector $k L/2 \pi$ and the difference between the lengths $L_1$ and $L_2$, namely $\delta$, where $\delta =(L_1-L_2)/2$. The considered parameters are $L_1=(100+\delta )$ nm, $L_2=(100-\delta )$ nm. For each pair of (*p*, *q*) and $n=0,1,2,...$, we get several values for $k L/2 \pi$ (Eq. 21) where BICs exist. For example, if $p=q=1$ and $\delta =0$, then the BICs will appear (using Eq. 21) at $k L/2 \pi = 1/2, 3/2, 5/2,...$. Another example is shown for $p=3, q=1$. The BICs appear (using Eq. 21) at $k L/2 \pi = 1, 3, 5,...$ and various values of $\delta$. The black circles represent the BIC associated to the pair $(p=3, q=1)$ and $n=2$, i.e., at $(k L/2 \pi , \delta )=(5,10)$. In order to give a better understanding about the behavior of the BICs, we will focus, in what follows, on the BIC associated to this pair.

Where $L'=L_0/2$, $T_p$ and $T_q$ are the Chebyshev polynomials of the first kind, and $C(L')=C(L_0/2)=Cos(k L_0/2)$. Since *p* and *q* are odd numbers, one can factorize $C(L')$ out of each term of the above equation, i.e. Eq. (19) can be written as

20 $$\begin{aligned} \begin{aligned} C(L') \{ S(p L') T'_q[C(L')] +S(q L') T'_p[C(L')] + i C(L') T'_p[C(L')] T'_q[C(L')] \} = 0, \end{aligned} \end{aligned}$$

where $T_p[C(L')]=C(L') T'_p[C(L')]$ and $T_q[C(L')]=C(L') T'_q[C(L')]$. Therefore the BIC states are given by $C(L')=Cos(k L')=0$, i.e.

21 $$\begin{aligned} \dfrac{k L}{2 \pi }= (2n+1) (p+q)/4, \end{aligned}$$

where $n=0, 1, 2,...$. Therefore for each pair of (*p*, *q*) we will have a BIC state (see Fig. 2).

### SIBIC states and their localizations

Another effect is that each loop may create one SIBIC state when a lead is attached to the port 0. Equation (12) (or equivalently Eq. 13) enables to conclude that for $C(L_1/2)=0$ and for $C(L_2/2)=0$ the eigenfunctions vanish on the port 0, which in turn induce transmission zeros. The one corresponding to $C(L_1/2)=0$ is a SIBIC state localized in one semi-infinite lead and in the loop $L_1$. Similarly, the one corresponding to $C(L_2/2)=0$ is a SIBIC state localized in one semi-infinite lead and in the loop $L_2$. When this happens for different eigen-wave vectors, this system has two SIBIC states. When this happens for the same wave vector, $L_1$ should be commensurable with $L_2$. In such case these SIBIC states may induce BIC state (see for example Fig. 2 and Eq. 17) for the case where $L_1=(11/9)L_2$). These SIBIC and BIC states induce transmission zeros (important for the creations of LLR).

Let us stress that for the eigenstates given by the zeros of the real part of the denominator of Eq. (13) ($S(L)=0$), we get $t=1$. So there are transmission ones between the transmission zeros provided by the $C(L_1/2)=0$ and the $C(L_2/2)=0$ SIBIC states. Therefore this system shows LLR for any incommensurate or commensurate values of $L_1$ and $L_2$.

**Fig. 3:**
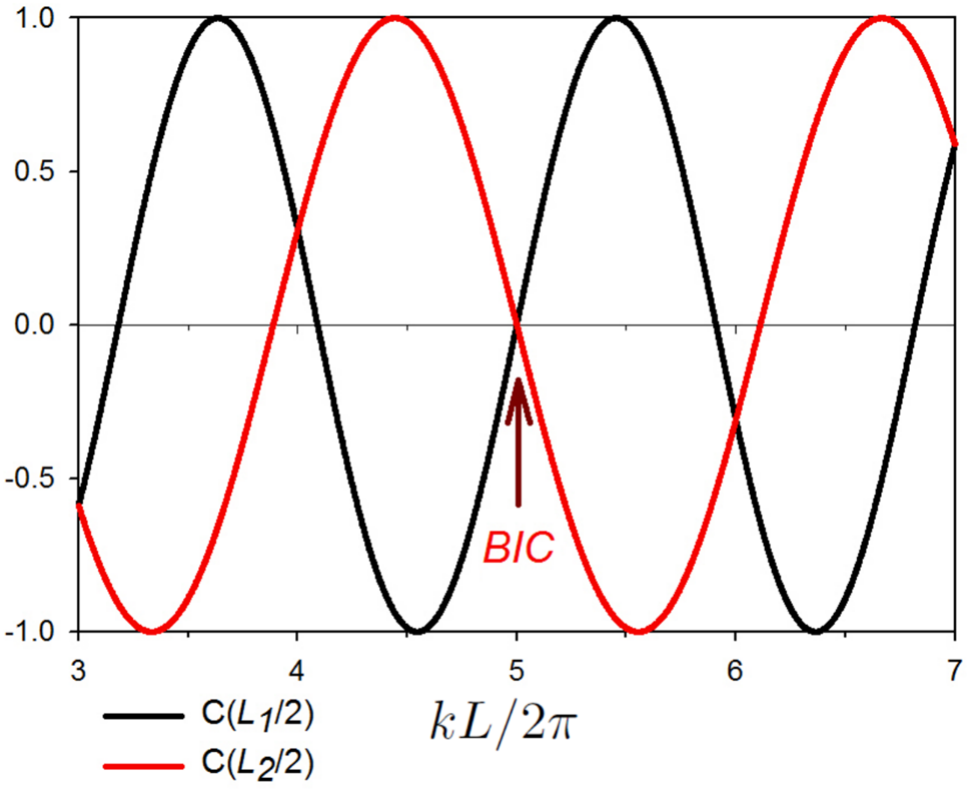
Illustrative example for the existence of the BIC states due to the intersection of the curves given by $C(L_1/2)=0$ and $C(L_2/2)=0$. The parameters used for this plot is $L_1=110$ nm, $L_2=(9/11)L_1=90$ nm. The BIC states appears (using Eqs. 17 and 21 for $p=3$, $q=1$) at $k L/2 \pi = 1, 3, 5,...$, see Fig. 2.

Figure 2a,b give, respectively, the variation of the transmittance and reflectance rates (with color scale) versus the reduced wave vector $k L/2 \pi$ and the difference between the lengths $L_1$ and $L_2$, namely $\delta$, where $\delta =(L_1-L_2)/2$. One can notice that for each pair (*p*, *q*) and $n=0,1,2,...$, BICs occur for $k L/2 \pi =(2n+1)(p+q)/4$ (Eq. 21). These modes (BICs) appear at the intersection of the curves given by $C(L_1/2)=0$ and $C(L_2/2)=0$ (see Fig. 3). One can also notice that at these points the transmittance (reflectance) is completely zero (one).

In fact, the BICs have robust zeros at port 0. In order to make these BIC states pop out as a sharp LLR when plotting the transmittance curve we have to break the symmetry of the structure shown in Fig. 1b by increasing/decreasing the loop length $L_1$ (or $L_2$). Figure 2a shows how the width of these resonances can be tuned using the length difference parameter $\delta$. The color code given on the right enables to understand how this resonance increase/decreases in function $k L/2 \pi$.

In order to give a better understanding about the behavior of the SIBICs, BICs and the associated Fano resonances in the transmittance amplitude, we will focus, in what follows, on the BIC associated to the pair $(p=3, q=1)$ and $n=2$, i.e., at $(k L/2 \pi , \delta )=(5,10)$, see Fig. 2a.

**Fig. 4:**
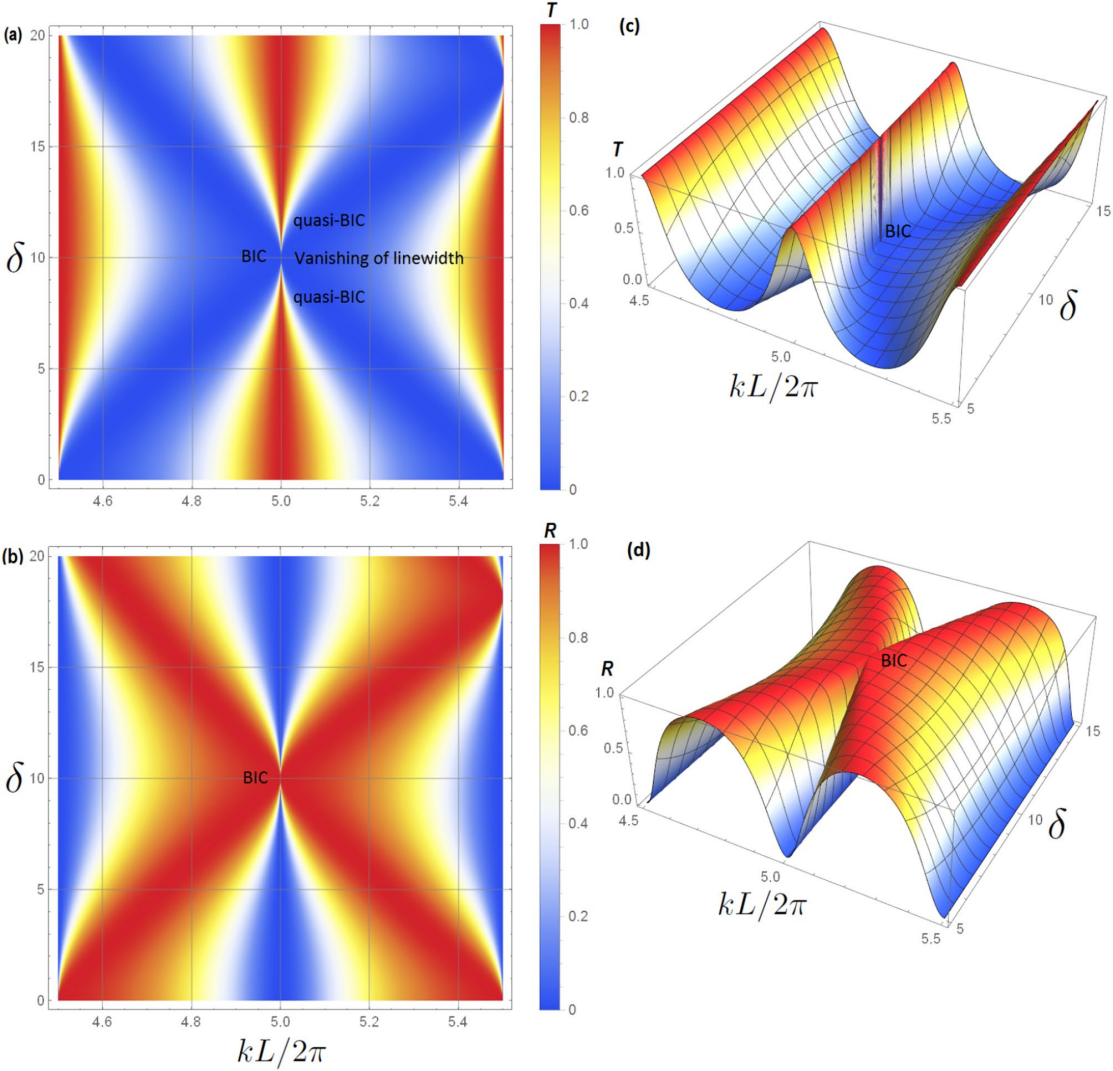
For the structure presented in Fig. 1b the above plots shows a zoom-in of the variation of transmittance (**a**) and reflectance (**b**) (with color scale) versus the reduced wave vector $k L/2 \pi$ and the length difference parameter $\delta$. The considered parameters are $L_1=(100+\delta )$ nm, $L_2=(100-\delta )$ nm. In this plot we are focusing on the BIC associated to the pair $(p=3, q=1)$ and $n=2$, i.e., at $(k L/2 \pi , \delta )=(5,10)$. One can notice the narrowing of the resonance of the quasi-BIC, then its transformation into a BIC (vanishing of linewidth) at $(k L/2 \pi , \delta )=(5,10)$. A transparency window between two zeros (induced by the SIBICs) appears when we deviate slightly from the BIC condition, giving rise to symmetric Fano resonance. (**c**) and (**d**) shows a three dimensional maps of the transmittance and reflectance given in (a) and (b) respectively.

In Fig. 4 we plot a zoom-in of the variation of transmittance (a) and reflectance (b) (appearing in Fig. 2) versus $k L/2 \pi$ and the length difference parameter $\delta$. The black circles in Fig. 2 represent the BIC position. One can notice the narrowing of the resonance of the quasi-BIC, then its transformation into a BIC (vanishing of linewidth) at $(k L/2 \pi , \delta )=(5,10)$. A transparency window between two zeros (induced by the SIBICs) appears when we deviate slightly from the BIC condition, giving rise to Fano resonance. Figure 4c,d shows a three dimensional maps of the transmittance and reflectance given in 4a,b respectively.

**Fig. 5:**
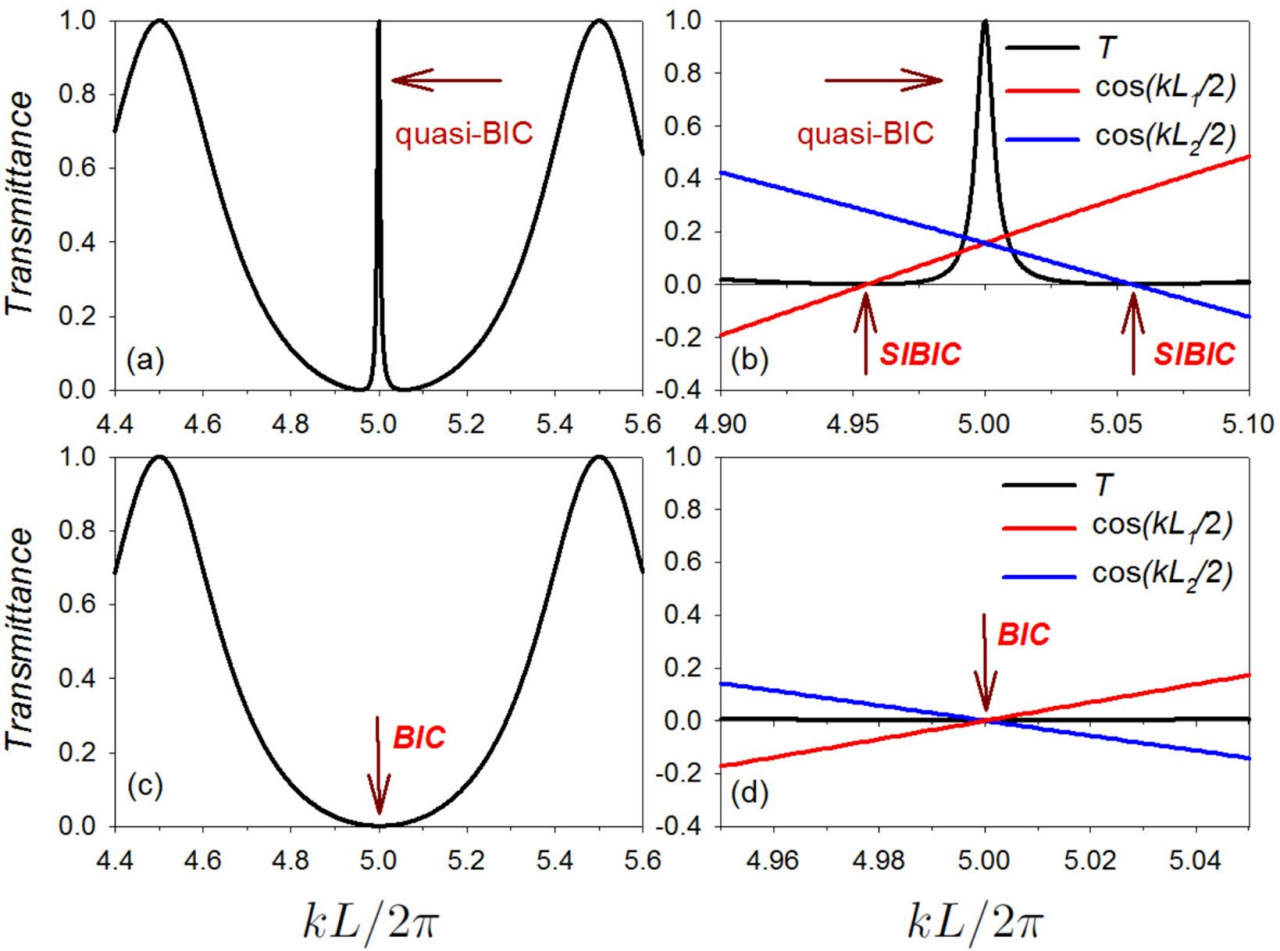
For the structure presented in Fig. 1b the plots in (**a**) and (**c**) shows the variation of transmittance versus the reduced wave vector $k L/2 \pi$ for two values of the length difference parameter $\delta$, namely $\delta =11$ nm and 10 nm respectively. The lengths of the two loops are considered to be $L_1=(100+\delta )$ nm and $L_2=(100-\delta )$ nm. In this figure we are focusing on the BIC associated to the pair $(p=3, q=1)$ and $n=2$, i.e., at $(k L/2 \pi , \delta )=(5,10)$ (see Fig. 4a). The peak between the transmission zeros given by the two SIBICs (provided by $C(L_1/2)=0$ and $C(L_2/2)=0$ and denoted by dark red arrows in (**b**)) gives rise to a well defined quasi-BIC resonance. This resonance becomes narrow as $\delta$ decreases down to 10 nm. At $\delta = 10$ nm the width of this resonance disappears, giving rise to BIC (denoted by dark red arrow in (**c**)) at $k L/2 \pi =5.0$. The plot in (**d**) is the same as the one drawn in (**b**) but for $\delta = 10$ nm. In this figure one can see how the resonance is collapsed giving rise to BIC (denoted by dark red arrow in (**d**)) at $k L/2 \pi =5$.

In addition we plotted in Fig. 5a,c the transmittance spectra in function of $k L/2 \pi$ for two values of the length difference parameter $\delta$ , namely $\delta =11$ nm and 10 nm respectively. The lengths of the two loops are considered to be $L_1=L+\delta =100+\delta$ nm and $L_2=L-\delta =100-\delta$ nm. The peak between the transmission zeros given by the two SIBICs (provided by $C(L_1/2)=0$ and $C(L_2/2)=0$ and denoted by dark red arrows in Fig. 5b) gives rise to a well-defined quasi-BIC resonance. This result can be qualified as Fano resonance (see below). This resonance becomes narrow as $\delta$ decreases down to 10 nm . At $\delta = 10$ nm the width of this resonance disappears, giving rise to BIC (denoted by dark red arrow in Fig. 5c) at $k L/2 \pi =5$. The plot in Fig. 5d is the same as the one drawn in Fig. 5b but for $\delta = 10$ nm. In this figure one can see how the resonance is collapsed giving rise to BIC (denoted by dark red arrow in Fig. 5d) at $k L/2 \pi =5$ . The BIC transforms to a quasi-BIC as we shift out from the BIC position. In general, the quasi-BIC manifests itself as a Fano resonance in the transmittance and a narrow resonance in the VADOS (see below).

**Fig. 6:**
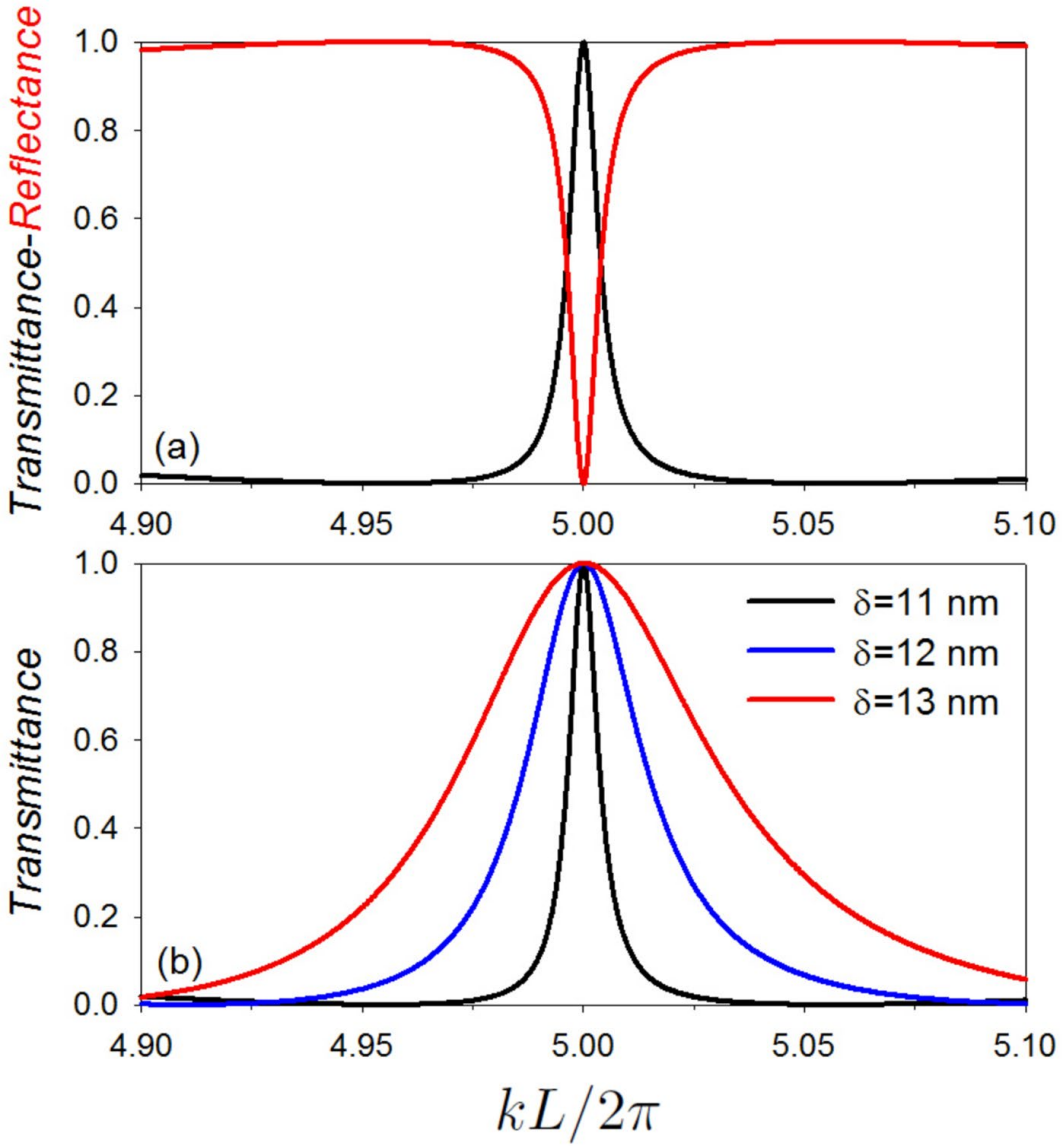
(**a**) A zoom-in picture for the transmittance (black line) and reflectance (red line) coefficients versus $k L/2 \pi$ for the resonance given in Fig. 5a (or 5b). The parameters are $\delta =11$ nm and $L_1=(100+\delta )$ nm, $L_2=(100-\delta )$ nm. Notice that the two loops interfere destructively (constructively), giving rise to a zero reflectance (total transmittance). This result is in accordance with the conservation law $R + T = 1$. Note also that the total transmittance occurs between two transmission zeros induced by the two SIBICs provided by $C(L_1/2)=0$ and $C(L_2/2)=0$. This result can be qualified as Fano resonance. (**b**) The same as the transmittance in (a) but for $\delta =11$ nm (black line), 12 nm (blue line) and 13 nm (red line ) respectively. One can notice that with increasing (decreasing) the value of the tuning parameter $\delta$ (i.e. the difference between the lengths of the two loops), the quality of the resonance is decreased (increased).

Figure 6a represents a zoom-in picture for the transmittance (black line) and reflectance (red line) coefficients versus the reduced wave vector $k L/2 \pi$ for the quasi-BIC resonance given in Fig. 5a (or 5b). The parameters are $\delta =11$ nm and $L_1=(100+\delta )$ nm, $L_2=(100-\delta )$ nm. One can notice that the two loops interfere destructively (constructively), giving rise to a zero reflectance (total transmittance). This result is in accordance with the conservation law $R + T = 1$ and the discussion given above where the total transmittance occurs between two transmission zeros induced by the two SIBICs provided by $C(L_1/2)=0$ and $C(L_2/2)=0$. The transmittance in Fig. 6b is the same as the transmittance in Fig. 6a but for $\delta =11$ nm (black line), 12 nm (blue line) and 13 nm (red line) respectively. One can notice that with increasing (decreasing) the value of the tuning parameter $\delta$ (i.e. the difference between the lengths of the two loops), the quality of the resonance is decreased (increased).

**Fig. 7:**
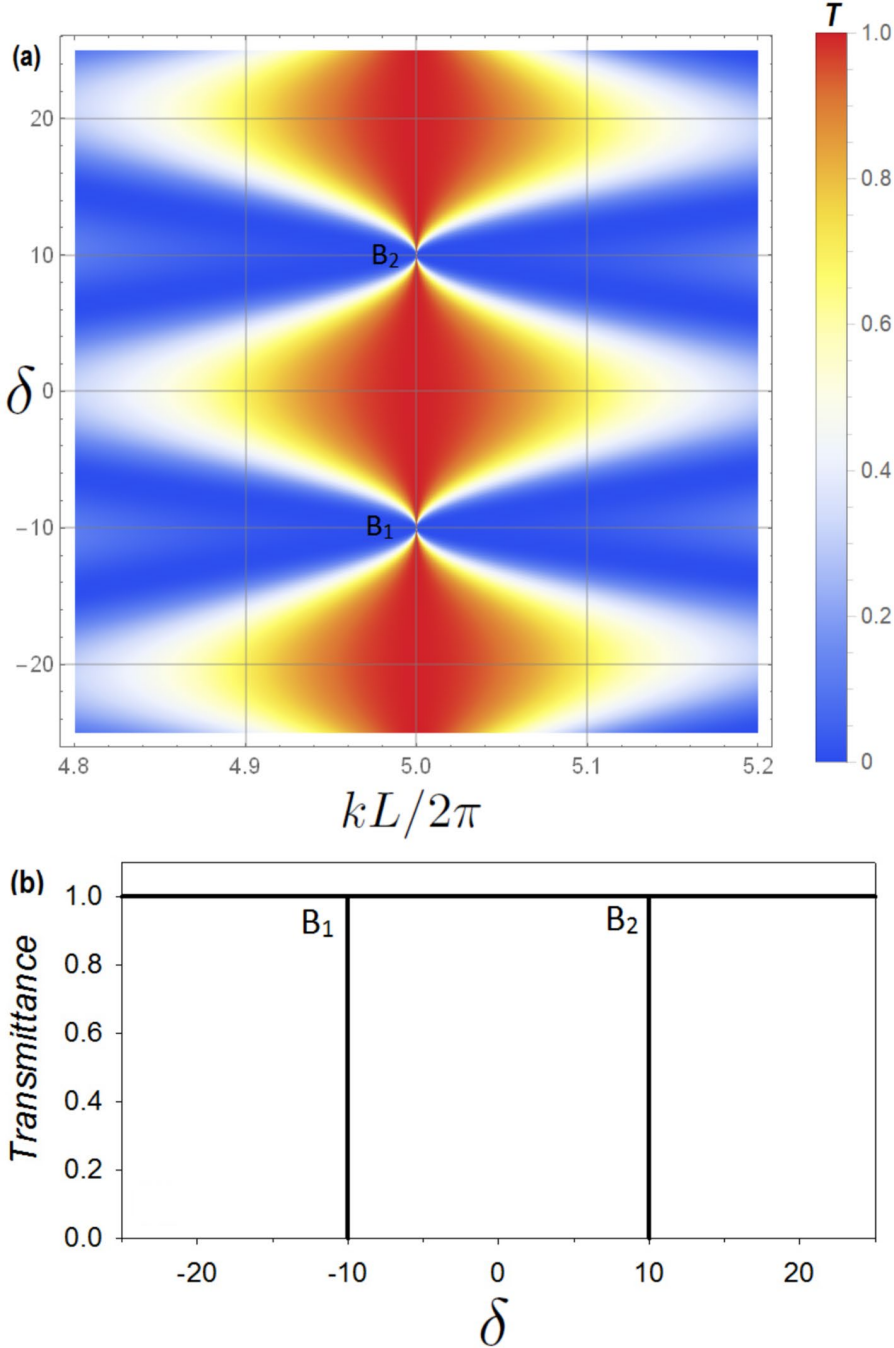
(**a**) shows a zoom-in of the transmittance variation (with color scale) versus the reduced wave vector $k L/2 \pi$ and the tuning parameter $\delta$. The parameters considered are $L_1=(100+\delta )$ nm, $L_2=(100-\delta )$ nm. In this plot we focus on the BICs that appear at $k L/2 \pi =5$. (**b**) Variation of the transmittance versus the tuning parameter $\delta$. The parameters considered are the same as in (a). The letters $B_1$ and $B_2$ appears in (**a**) and (**b**) showing the correspondence between the BICs appearing in (a) and the transmittance zeros appearing in (b).

Figure 7a shows a zoom-in of the transmittance variation (with color scale) versus the reduced wave vector $k L/2 \pi$ and the tuning parameter $\delta$. The parameters considered are $L_1=(100+\delta )$ nm, $L_2=(100-\delta )$ nm. In this plot, we focus on the BICs that appear at $k L/2 \pi =5$. In Fig. 7b we show the variation of the transmittance versus the tuning parameter $\delta$. The parameters considered are the same as in (a). The letters $B_1$ and $B_2$ appears in (a) and (b), showing the correspondence between the BICs appearing in (a) and the transmission zeros appearing in (b). These results show that the transmission zeros (total reflectance) can be controlled by detuning the lengths of the two loops.

### Fano resonance

**Fig. 8:**
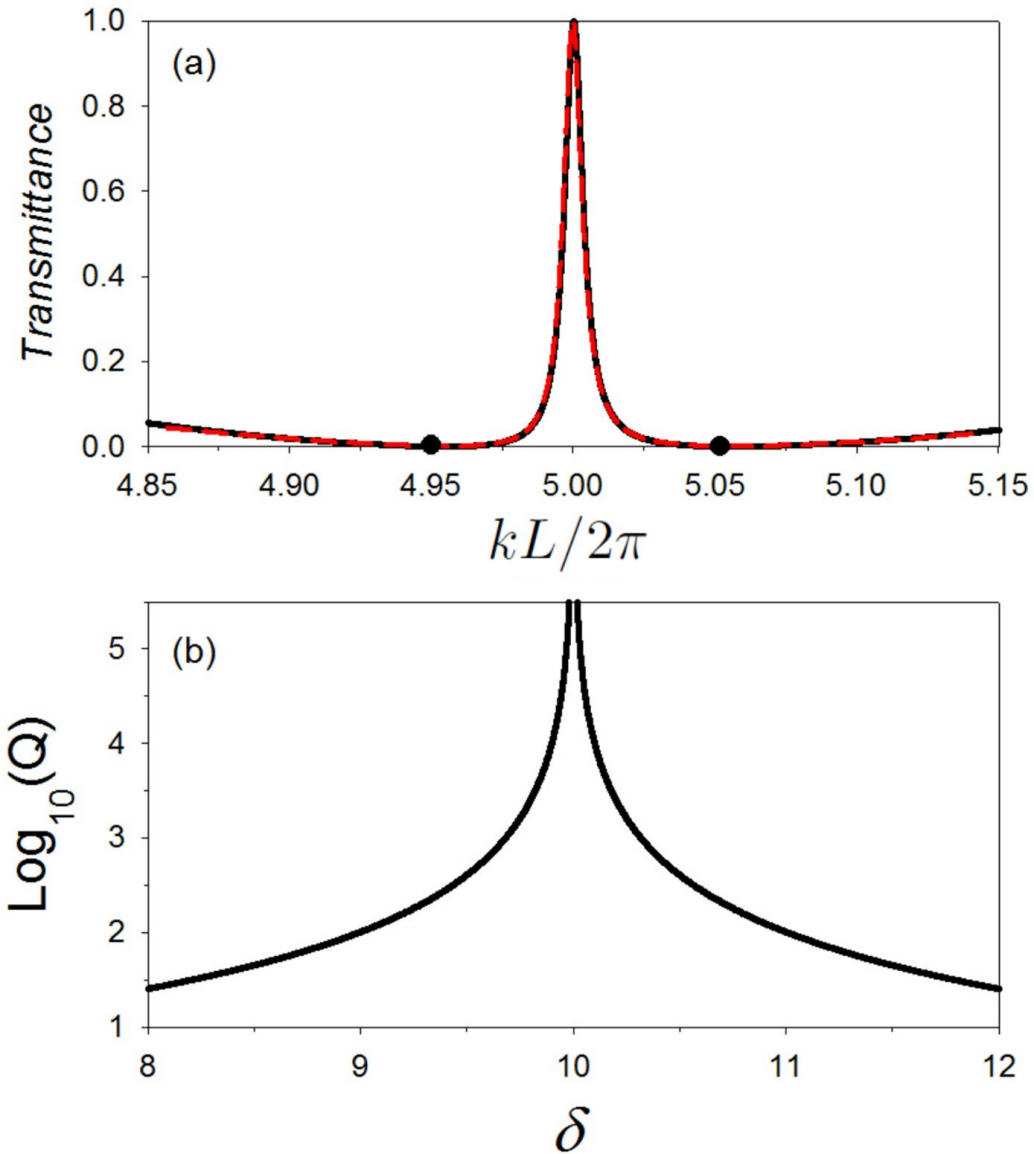
(**a**) The solid line presents a zoom-in plot for the resonance given in Fig. 6b with $\delta =11$ nm. The dashed red line presents the approximate expression Eq. (23) for $\Delta =0.05 \pi$. The approximate results are in a very good agreement with the exact ones (solid lines) and clearly show that the resonance is of Fano type with $q_1\simeq 11.47$, $q_2\simeq 14.31$ and width $2\Gamma \simeq 0.049348$. (**b**) variation of the logarithm of the quality factor *Q* of the peak reported in (a) as a function of the tuning parameter $\delta$. One can notice that the quality factor of the peak depends on the value of $\delta$ and as predicted diverges when $\delta =10$ nm.

The resonance in Fig. 5a shows the same characteristics as a Fano resonance but with two zeros (due to the two SIBICs) of transmission around the resonance instead of one, as is usually the case. Indeed, one can obtain an approximate analytical expression for the transmission function (Eq. 13) in the vicinity of the resonance. A Taylor expansion around $k L/2 \pi =5.0$ (i.e. $k L/2 \pi =5.0 + \varepsilon /2\pi$ with $\varepsilon /2\pi \ll 1$ ) enables us to write the transmission function (Eq. 13) as

22 $$\begin{aligned} t = \frac{-iA \zeta \zeta '}{\varepsilon -iA \zeta \zeta '}. \end{aligned}$$

where $A=\frac{L_1L_2}{4 L^2}$, $\zeta =\varepsilon +2\Delta \frac{L}{L_1}$, $\zeta '=\varepsilon -2\Delta \frac{L}{L_2}$ and $\Delta$ is the detuning of $L_1/L$ and $L_2/L$ from 11/10 and 9/10 respectively (i.e., $\Delta =\pi (\frac{L_1}{L}-\frac{11}{10})=\pi (\frac{9}{10}-\frac{L_2}{L})$). From Eq. (22), one can show that the transmittance *T* can be written following the Fano line shape^8^ in the form

23 $$\begin{aligned} T = A^2 \frac{(\varepsilon +q_1 \Gamma )^2(\varepsilon -q_2 \Gamma )^2}{\varepsilon ^2 +\Gamma ^2}. \end{aligned}$$

where $q_1=\frac{2L}{\Delta L_1}$ and $q_2=\frac{2L}{\Delta L_2}$ are the Fano parameters^66,67^, and $\Gamma =\Delta ^2$. Equation (23) shows that the transmission vanishes at two approximate values given by $\varepsilon _1 =- q_1 \Gamma$ and $\varepsilon _2 = q_2 \Gamma$. One can also notice that $q_1$ and $q_2$ increases when $\Delta$ decreases and tends to infinity when $\Delta$ vanishes. In this case, the resonance falls at $\varepsilon =0.0$ and its width $2\Gamma$ reduces to zero as expected. The results of the approximate expression Eq. (23) are sketched (dashed red lines) in Fig. 8a for $\Delta =0.05 \pi$. These results are in a very good accordance with the exact ones (solid lines) and clearly show that the resonance is of Fano type with $q_1\simeq 11.47$, $q_2\simeq 14.31$ and width $2\Gamma \simeq 0.049348$.

In Fig. 8b, we have displayed the variation of the logarithm of the quality factor *Q* of the peak reported in Fig. 8a as a function of the tuning parameter $\delta$. The quality factor *Q* is defined as the ratio between the central frequency and the full width at half maximum ($Q= 5.0/2\Gamma$). One can notice that the quality factor of the peak is inversely proportional to the square of the detuning parameter $\Delta$ and in turn, depends on the value of $\delta$ and as predicted diverges when $\delta =10$ nm. This result enable us to increase the quality factor of the peaks to infinite values by detuning the lengths of the two loops $L_1$ and $L_2$. This property is a feature of Fano and induced transparency resonances that does not exist in standard waveguide structures with defect^16,68^. It should be pointed out that the validity of our results is subject to the requirement that the cross-section of the guides is negligible compared to their length and to the propagation wavelength.

In fact, while Fano resonances and other interference-based phenomena have been widely explored for sensing and filtering applications, several challenges remain in achieving optimal performance for practical devices. For example, many existing implementations of Fano-resonant systems suffer from a tradeoff between sensitivity and resonance linewidth^69^. High sensitivity often comes at the cost of broader linewidths (lower Q-factors), which limits spectral resolution in sensing and filtering. In contrast, the LLR demonstrated in our cavity structure exhibit very high Q-factors (Fig. 8b), enabling more precise detection of weak perturbations.

## Transmission phase and state phase shift

The transmission phase is obtained from Eq. (13) to be

24 $$\begin{aligned} \phi =\tan ^{-1}\left( \frac{S (L)}{C \left( { L_1}/{2}\right) C ( L_2/2)}\right) . \end{aligned}$$

Equation (22) also enables us to deduce an approximate expression for the phase as

25 $$\begin{aligned} \phi =\tan ^{-1}\left( \frac{-\varepsilon }{A \zeta \zeta '}\right) . \end{aligned}$$

Another interesting quantity is the first derivative of $\phi$ with respect to the energy, which is related to the delay time taken by the electrons to traverse the structure. This quantity, called phase time, is defined by^70^

26 $$\begin{aligned} \tau _\phi =\hbar \frac{d\phi }{d E}. \end{aligned}$$

Moreover, another interesting entity that can be extracted from the Green’s function is the bulk state phase shift $\eta$. This bulk state phase shift between the final system (the two loops with the leads) and the reference system (the isolated loops and the two semi-infinite leads) is given by^62^

27 $$\begin{aligned} \eta =-\text {arg}\left[ \text {det}\left\{ g^{-1}(MM)\right\} \right] . \end{aligned}$$

From Eq. (12) one can deduce that

28 $$\begin{aligned} \eta =-\tan ^{-1}\left( \frac{ C \left( { L_1}/{2}\right) C ( L_2/2)}{S ( L)}\right) . \end{aligned}$$

Using Eq. (25) one can write an approximate expression for the bulk state phase shift as

29 $$\begin{aligned} \eta =\tan ^{-1}\left( \frac{A \zeta \zeta '}{\varepsilon }\right) . \end{aligned}$$

In order to provide an analytical comparison of the density of states with the phases involved in the system, we consider the variation of the density of states (VADOS) $\Delta n(E)$ between the final system depicted in Fig. 1b and the reference system composed of the two loops and the two semi-infinite leads. This quantity is given by^62^

30 $$\begin{aligned} \Delta n (E)=- \frac{\hbar }{\pi } \frac{d \eta }{d E}. \end{aligned}$$

Note that the $\pi$ drops in $\phi$ and $\eta$ are due to the zero values of the denominators appearing in their respective analytical expressions. As these denominators are not the same, the $\eta$ and $\phi$, $\pi$ drop positions are not the same.

**Fig. 9:**
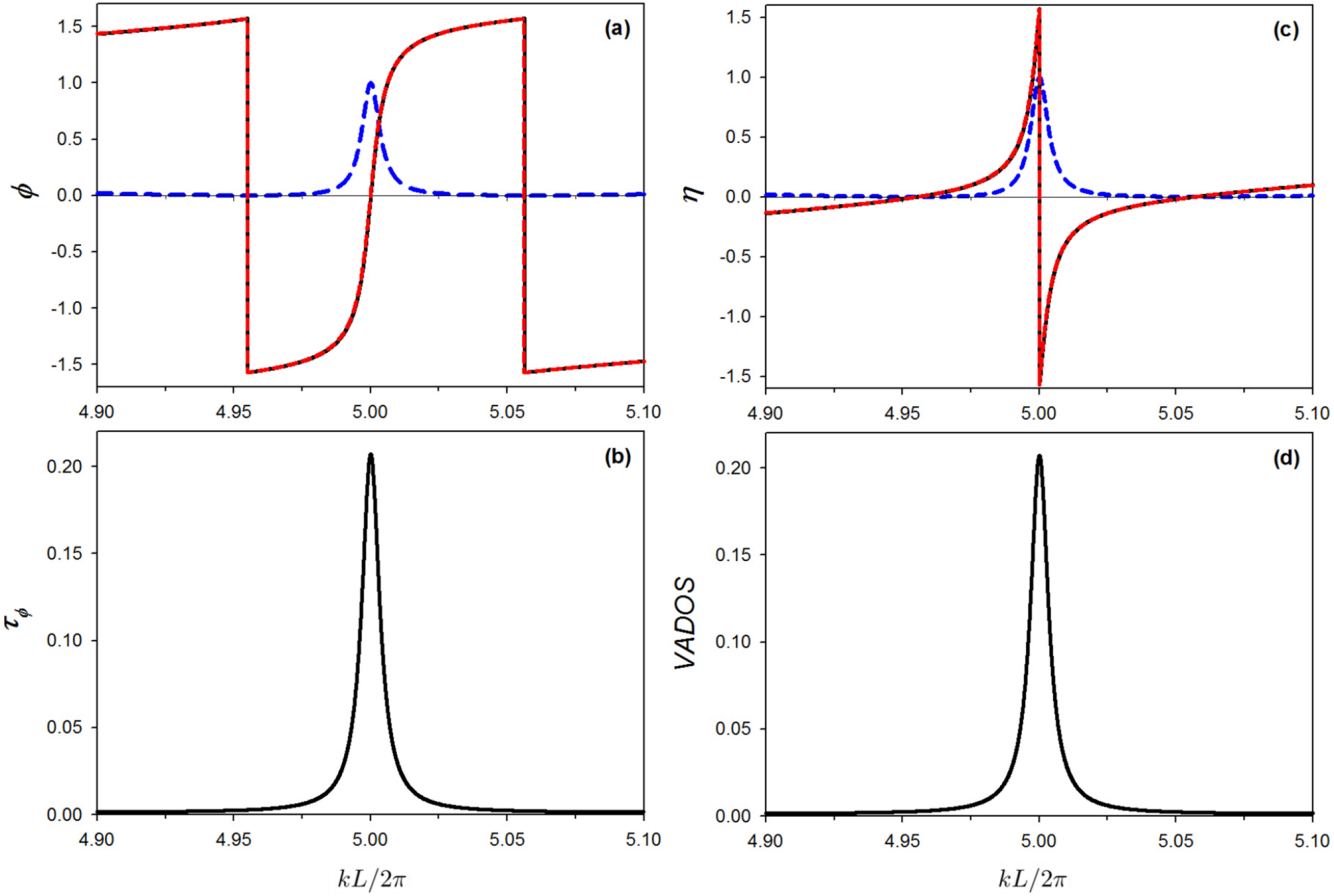
(**a**) Transmission phase versus $k L/2 \pi$ for the structure presented in Fig. 1b with $L_1=100+\delta$ nm, $L_2=100-\delta$ nm and $\delta =11$ nm. The blue dashed curve recalls the transmittance curve. The $\pi$ drops at $k L/2 \pi \simeq 4.95495$ and 5.05618 are induced by $C(L_1/2)=0$ and $C(L_2/2)=0$ SIBIC states respectively. The approximate function for the transmission phase given in Eq. (25) is plotted by red dashed lines and clearly show two abrupt phase change of $\pi$ at $\zeta =0$ and $\zeta '=0$ (i.e. $\varepsilon =-2\Delta \frac{L}{L_1}$, $\varepsilon =2\Delta \frac{L}{L_2}$), in accordance with the exact result (solid line). The transmission phase exhibits a phase jump at the transmission zeros. This provides one single positive peak and two negative delta ones at $k L/2 \pi \simeq 4.95495$ and 5.05618 (not shown in the plot), in the phase time plot. (**b**) The phase time (in units of $2mL^2/\hbar$) versus $k L/2 \pi$. (**c**) The same as in (a) but for the bulk state phase shift. The $\pi$ drop (at $k L/2 \pi = 5.0$) is due to the loss of one bulk state induced by $S(L)=0$. This $\pi$ drop is associated with a maximum in the transmittance curve (Eq. 14) in blue dashed-plot, superposed here for agreement check. The approximate function for the bulk state phase shift given in Eq. (29) is plotted by red dashed lines and clearly show an abrupt phase change of $\pi$ at $\varepsilon =0$, in accordance with the exact result (solid line). (**d**) The same as in (b) but for the VADOS.

Figure 9a shows the transmission phase versus the reduced wave vector $k L/2 \pi$ for the structure presented in Fig. 1b with $L_1=100+\delta$ nm, $L_2=100-\delta$ nm and $\delta =11$ nm. The blue dashed curve recalls the transmittance curve. The $\pi$ drops at $k L/2 \pi \simeq 4.95495$ and 5.05618 are induced by $C(L_1/2)=0$ and $C(L_2/2)=0$ SIBIC states respectively. The approximate function for the transmission phase given in Eq. (25) is plotted by red dashed lines in Fig. 9a and clearly show two abrupt phase change of $\pi$ at $\zeta =0$ and $\zeta '=0$ (i.e. $\varepsilon =-2\Delta \frac{L}{ L_1}$, $\varepsilon =2\Delta \frac{L}{ L_2}$), in accordance with the exact result (solid line). The transmission phase exhibits, a phase jump at the transmission zeros. This provides one single positive peak and two negative delta ones at $k L/2 \pi \simeq 4.95495$ and 5.05618 (not shown in the plot), in the phase time. Figure 9b shows the phase time (in units of $2mL^2/\hbar$) versus $k L/2 \pi$.

In Fig. 9c we plot the bulk state phase shift versus the reduced wave vector $k L/2 \pi$ for the structure presented in Fig. 1b with $L_1=100+\delta$ nm, $L_2=100-\delta$ nm and $\delta =11$ nm. The $\pi$ drop (at $k L/2 \pi = 5.0$) is due to the loss of one bulk state induced by $S(L)=0$. This $\pi$ drop is associated with a maximum in the transmittance curve (Eq. 14) in blue dashed-plot, superposed here for agreement check. The approximate function for the bulk state phase shift given in Eq. (29) is plotted by red dashed lines in Fig. 9c and clearly show an abrupt phase change of $\pi$ at $\varepsilon =0$, in accordance with the exact result (solid line). Figure 9d shows the VADOS versus $k L/2 \pi$. Let us mention that if we introduced the dissipation in the system by adding a small imaginary part to the energy *E*, i.e., *E* becomes $E \pm i(0.0001)$ a negative delta peak (not shown in the plot) will show up in the VADOS plot due to the loss of the bulk state at $k L/2 \pi = 5.0$. The phase time and the VADOS are exactly the same, when one neglects the derivatives of the $\pi$ drops. This happens only when one has two leads.

## Two tangent loop cavity with one side semi-infinite lead

**Fig. 10:**
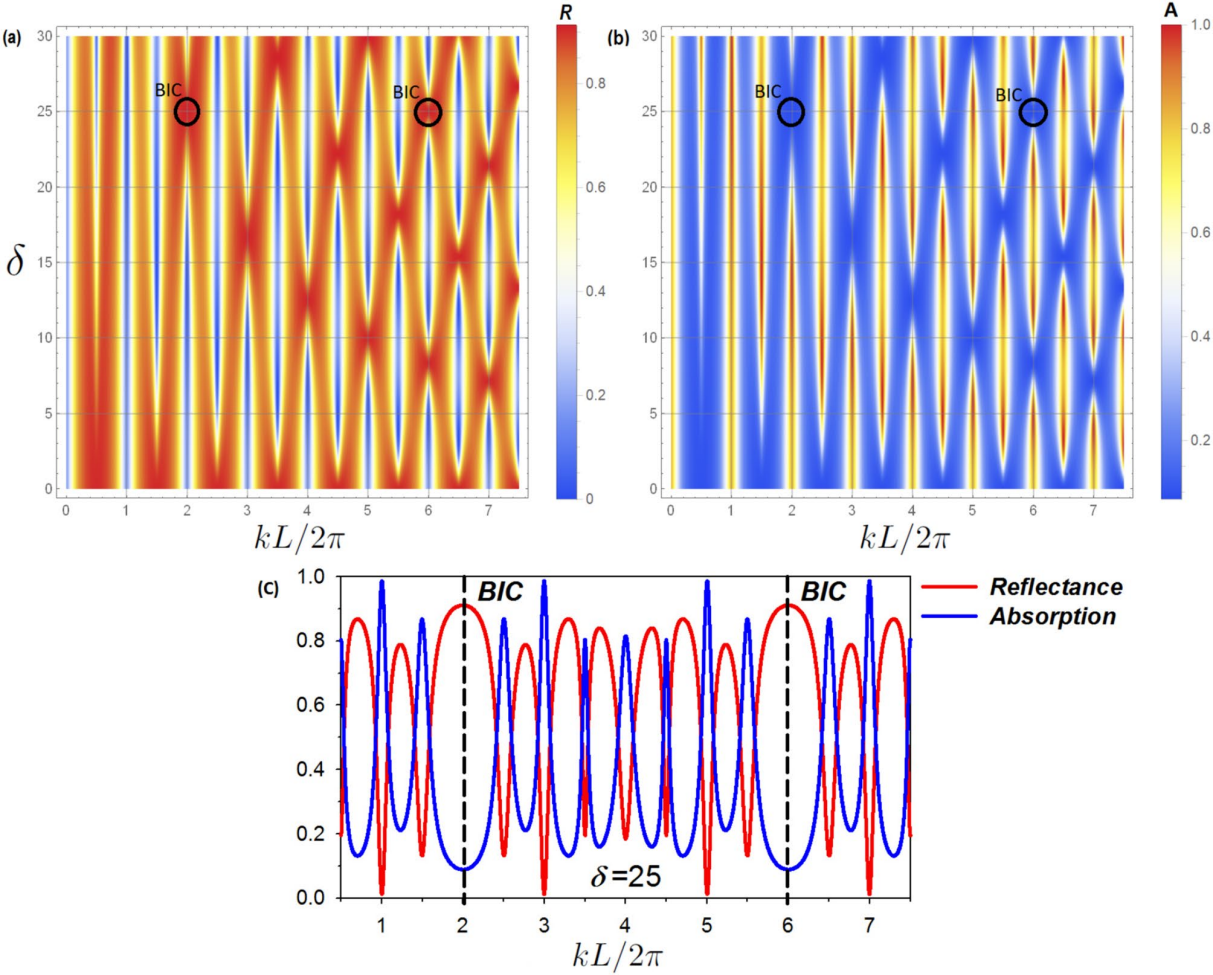
For the electronic cavity structure presented in Fig. 1c the above plots shows the variation of reflectance (**a**) and absorption (**b**) (with color scale) versus the reduced wave vector $k L/2 \pi$ and the difference between the lengths $L_1$ and $L_2$, namely $\delta$. The considered parameters are $L_1=(100+\delta )$ nm, $L_2=(100-\delta )$ nm. These figures reveals the behavior of different BICs and resonances of the cavity. Indeed, the BICs already discussed in Figs. 2 and 4 appear here also. The black circles represent the BICs associated to $(k L/2 \pi , \delta )=(2,25)$ and (6, 25). (**c**) Variation of reflectance (red curve) and absorption (blue curve) spectra vs $k L/2 \pi$ for $\delta =25$ nm. Black dashed lines indicate the BICs positions.

In this section we study the case where the two loops cavity are in contact with only one semi-infinite guide attached to the port from the left side (see Fig. 1c). The different modes in the cavity can be investigated by means of the reflection coefficient given by

31 $$\begin{aligned} r = \frac{2S(L)-iC(L_1/2)C(L_2/2)}{2S(L) +i C(L_1/2)C(L_2/2)}. \end{aligned}$$

It is worth mentioning that, in a perfect lossless system, the reflectance amplitude reaches unity ($R = |r|^2 = 1$), while in a real system, owing to the absorption, $R = 1-A$, where *A* is the absorption intensity. The reflection intensity provides different resonant modes of the cavity, including BICs. Indeed, due to the loss, the reflectance rate does not reach unity, and the resonant modes of the system appear as dips in the reflectance spectra. For a lossy system, Fig. 10 reveals the behavior of different BICs and resonances of the cavity, in the reflectance (Fig. 10a), and absorption (Fig. 10b), spectra as a function of $kL/2\pi$ and $\delta$. Indeed, the BICs already discussed in Figs. 2 and 4 appear here also.

As earlier demonstrated (subsection BIC States), the disappearance of the resonance at $\delta = 10$ nm and $kL/2\pi =5$ (Eq. 21) indicates the presence of a BIC. Likewise, when $\delta = 25$ nm and $kL/2\pi =2$ we observe a hidden resonance around $kL/2\pi =2$ for $L_1 = 125$ nm and $L_2 = 75$ nm; this behavior indicates the presence of BIC. Figure 10c depict the behavior of reflectance (red curve) and absorption (blue curve) through the lossy system as a function of $kL/2\pi$ for $\delta =25$ nm. The reflectance resonance tends to zero around $kL/2\pi =1, 3, 5,...$, whereas the absorption reaches a maximum value of $A \simeq 0.99$. Furthermore, around $kL/2\pi =2$ the intensity of this reflectance resonance increases, leading to the formation of a BIC at $\delta =25$ nm. This mode appears as a hidden resonance with a zero width, giving rise to a plateau in the reflectance ($R \simeq 0.9$), while the absorption approaches zero ($A \simeq 0.1$). Similar analysis can be done for the resonance appearing around $kL/2\pi =6$. These modes are indicated by vertical dashed lines in Fig. 10c. Let us mention that these hidden resonances can reappear by slightly detuning the lengths $L_1$ and $L_2$ from the BIC position.

Moreover, let us point out the possibility to reach a near-perfect absorption^71^ for some frequencies, as shown in Fig. 10c, where the wave penetrates the cavity without back reflection. In addition to the reflection intensity, the reflection delay time and VADOS can also be useful for analyzing the existence and behavior of the BICs in the two loops cavity.

## Discussion and conclusions

In summary, we have given an analytical evidence about the existence and behavior of SIBICs, BICs and Fano resonances in a 1D monomode electronic cavity structure made of two closed loops of lengths $L_1$ and $L_2$. The cavity can either be placed between two semi-infinite leads from both sides or just one semi-infinite lead from one side. In the case of a symmetric cavity inserted between two semi-infinite leads, we have demonstrated the condition of commensurability that should be satisfied by $L_1$ and $L_2$ as well as the corresponding reduced wave-vectors to get a BICs. A theoretical investigation of the electronic transmittance (reflectance) power using a Green’s function method is presented. Numerical results on sharp peaks in detuned waveguide were also reported. These peaks appear as Fano resonances of strong amplitude in the transmittance spectra and phase time. By considering an additional configuration where the two-tangent loops cavity is attached with one port from only one side, BICs can also be observed inside the structure. Such a cavity can be designed to realize near-perfect absorption for some frequencies.

The cavity structure proposed in this work exhibit the possibility to tune the quality factors of the induced resonances close to infinity by detuning the lengths $L_1$ and $L_2$. This property is a feature of Fano resonances and does not exist in standard waveguides with defect^16^. Let us also mention that the analogy between the Schrödinger equation and the Helmholtz equation for electromagnetic waves enables one to correlate the results for optical experiments to that for electrons. Therefore, Fano resonances can be understood in the same manner as for electromagnetic waves.

The BICs, induced by the symmetric cavity, discussed in this work are related to Fabry-Perot-type BICs commonly observed in optics. FP-BICs refer to destructive interference when two resonant cavities are spaced apart so that they are tuned to make the round-trip phase shift add up to an integer multiple of $2\pi$, causing destructive interference between the two resonances and then the formation of a BIC^72,73^. In fact, Fabry-Perot type BICs in both photonic and electronic systems relies on the same interference-based localization mechanism. They offer high confinement and are promising in resonance-based devices. However, they differ due to the type of waves involved, the materials used, and the way of coupling.

Both optical and electronic BICs share the property of having a resonance embedded in a continuous spectrum of modes, but they differ in their physical mechanisms, boundary conditions, and experimental realizability^7,13,22^. (i) The mechanism behind the existence of optical BICs is the interference between different resonant modes that leads to destructive interference, effectively preventing radiation and trapping light in certain regions of the structure. For electronic BICs quantum confinement effects (where the size of the structure or the boundary conditions of the system) leads to suppression of the interaction between the electronic wave-functions and the environment and in turn influence the electronic states to be bound in the continuum. (ii) Optical BICs are strongly dependent on the boundary conditions of the structure. These may include the periodicity (geometry) of the system (e.g., a photonic crystal or dielectric waveguide), the refractive index contrasts between materials, and the symmetry of the structure. For electronic BICs, boundary conditions are set by the potential landscapes, symmetry of states and electronic confinement. (iii) Optical BICs are experimentally realizable and have been demonstrated in a variety of optical systems. Photonic crystals and metasurfaces are two prominent platforms where optical BICs are studied. These systems allow the precise control of light-matter interactions. They have applications in light trapping, sensing, and lasers. Electronic BICs are harder to realize due to the fine control required over quantum states. However, they have been demonstrated in quantum dots and semiconductor hetero-structures. Finally let us mention that optical properties can be easier to adjust (refractive index, geometry) to achieve BICs, making their experimental realization more straightforward compared to electronic BICs.

In fact the majority of current studies are done with topological simulation approaches focusing on small deformations and one BIC state. Although introducing the state phase within the numerical routines is not trivial, this is expected to complete and improve simulation results. The knowledge of all system BIC and SIBICs, rather than only one, enables to choose the better one for a given application (a complete study of SIBICs is presented in our previous work^2,61^). This may help also to use several degenerate strictly bound states for novel systems. It is worth mentioning that in our previous work^74^, we presented a theoretical demonstration of BICs in an asymmetric loop composed of two arms of lengths $L_1$ and $L_2$ with both an experimental validation in the radio-frequency (RF) domain using coaxial cables and a numerical validation in the infrared (IR) domain using plasmonic metal-insulator-metal nanometric waveguides. The analytical study is performed by means of the Green’s function method, whereas the numerical calculation is obtained using COMSOL. The obtained results corroborate well with those obtained using the Green’s function formalism.

Finally, it is worth mentioning that in the proposed work we have dealt with an infinitesimally thin (1D) waveguide where the cross section of the wires are not taken into account. This leads us to expect that, the experimental work on a relatively thick wire may lead to results that are slightly different than ours.

## Data Availability

The data used and/or analyzed during the current study are available from the corresponding author on reasonable request.
